# Reconstituted B cell receptor signaling reveals carbohydrate-dependent mode of activation

**DOI:** 10.1038/srep36298

**Published:** 2016-10-31

**Authors:** Rina F. Villar, Jinal Patel, Grant C. Weaver, Masaru Kanekiyo, Adam K. Wheatley, Hadi M. Yassine, Catherine E. Costello, Kevin B. Chandler, Patrick. M. McTamney, Gary J. Nabel, Adrian B. McDermott, John R. Mascola, Steven A. Carr, Daniel Lingwood

**Affiliations:** 1 The Ragon Institute of Massachusetts General Hospital, The Massachusetts Institute of Technology and Harvard University, 400 Technology Square, Cambridge, MA 02139, USA; 2 The Broad Institute of The Massachusetts Institute of Technology and Harvard University, 415 Main St, Cambridge, MA 02142, USA; 3 Vaccine Research Center, National Institute for Allergy and Infectious Diseases, National Institutes of Health, 40 Convent Drive, Bethesda, MD 20814, USA; 4 Department of Microbiology and Immunology, University of Melbourne, Peter Doherty Institute for Infection & Immunity, 792 Elizabeth Street, Melbourne, VIC 3000, Australia; 5 Qatar University Biomedical Research Center, P.O. Box 2713-Doha-Qatar, QU-NRC, Qatar; 6 Boston University School of Medicine, 670 Albany Street, Boston MA 02118-2646, USA; 7 Medimmune, LLC, One MedImmune Way, Gaithersburg, MD 20878, USA; 8 Sanofi, 640 Memorial Drive, Cambridge, MA 02139, USA.

## Abstract

Activation of immune cells (but not B cells) with lectins is widely known. We used the structurally defined interaction between influenza hemagglutinin (HA) and its cell surface receptor sialic acid (SA) to identify a B cell receptor (BCR) activation modality that proceeded through non-cognate interactions with antigen. Using a new approach to reconstitute antigen-receptor interactions in a human reporter B cell line, we found that sequence-defined BCRs from the human germline repertoire could be triggered by both complementarity to influenza HA and a separate mode of signaling that relied on multivalent ligation of BCR sialyl-oligosaccharide. The latter suggested a new mechanism for priming naïve B cell responses and manifested as the induction of SA-dependent pan-activation by peripheral blood B cells. BCR crosslinking in the absence of complementarity is a superantigen effect induced by some microbial products to subvert production of antigen-specific immune responses. B cell superantigen activity through affinity for BCR carbohydrate is discussed.

The antibody response differs from innate immune recognition in that there is no pre-encoded specificity for antigen. Antigen is initially perceived by the complementarity loops (CDRs) of the germline B cell receptor (BCR). Each naïve B cell displays a unique CDR configuration that has been stochastically reconfigured to provide a diversity of potential antigen binding partners^1^,^2^,^3^,^4^. If recognition occurs then multivalent presentation of the antigen’s target epitope will induce BCR receptor signaling and cellular activation, gating the initiation of the antibody response^5^,^6^. Naïve B cells also display complement receptors that can recognize complement-decorated antigen to enhance BCR complementarity^7^,^8^, and activation can be further co-stimulated by TLR signaling pathways, providing additional adjuvancy during this first signaling phase^9^,^10^. Less understood is the role of cell surface carbohydrate. Lectins have been long described to initiate T cell signaling^11^,^12^,^13^,^14^,^15^, however, relatively little has been described for B cells. Glycan on the antigen surface can modulate both antibody complementarity^16^,^17^ and Siglec-based modulation of BCR output^18^,^19^, however, minimal attention has been given to glycan structures on the BCR itself. The BCR is highly glycosylated^20^, and one relevant historic observation is that ‘exotic’ sialic acid (SA)-binding lectins isolated from lobster can selectively stimulate mammalian B cells in the absence of antigen specificity^21^. Host surface sialyl-oligosaccharide is also the primary receptor for a number of enveloped viruses^22^,^23^, with the affinity of the influenza spike protein hemagglutinin (HA) for cellular SA being both a structurally-defined example and one of the most extensively characterized glycan-protein interactions^24^,^25^,^26^,^27^. In this study, we used influenza lectin activity for sialyl-oligosaccharide as a structurally-defined tool to define whether antigen affinity for SA represented a modality through which BCR activity may be tuned. Employing a new approach to reconstituting interactions between antigen and sequence-defined BCRs, we demonstrated that the germline receptor signals through both CDR driven antigen complementarity and non-cognate interactions provided by antigen affinity to SA. The latter was dependent on multivalent ligation of BCR sialyl-oligosaccharide and was manifested as pan-activation of naïve peripheral blood B cells. Pan B cell activation is a hallmark of superantigen activity, wherein antigen specific responses are depressed by non-cognate ligation of available BCR^28^,^29^. Induction of superantigen activity through a viral lectin affinity for BCR SA is discussed.

## Results

### A structurally defined SA-binding reagent

**Figure 1:**
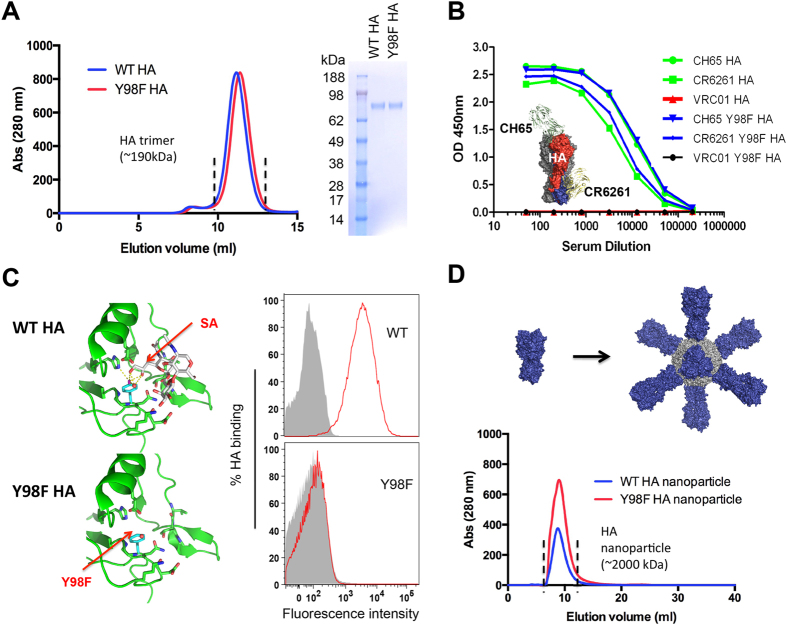
Tyr98-dependent binding of HA to SA provides a structurally defined multivalent affinity for this carbohydrate sequence. (**A**) Both WT HA and Y98F HA are the correct size of the HA trimer as defined by size exclusion on a Sepharose 10/300 column and are electrophoretically identical. (**B**) HA trimer or Y98F HA were also antigenically identical as measured by reactivity to two conformational antibodies: the HA receptor binding site specific antibody CH65 and the HA stem-specific antibody CR6261. Reactivity of the HIV-specific antibody VRC01 is presented as an isotype control. The HA antigen in complex with CH65 and CR6261 is derived from 3SM5 and 3GBM entries in the PDB. Surface plasmon resonance has also shown that for CH65 and CR6261, the association/dissociation rates and affinities for this antigen are unaffected by removal of the oxygen atom at amino acid position 98^34^. (**C**) The crystal structure displaying the HA loops with residues making hydrogen bond interactions with the SA (displayed by receptor analog LSTc l^26^, PDB 1RVZ). Polar contacts are shown as dashed lines, a water molecule is in red, and Try 98 is in pink (hydroxyl group) and cyan, respectively. When the hydroxyl group is removed through Y98F, SA-binding is lost^24^,^25^,^34^, as depicted by Tyr98 dependent adherence to 293F cells. (**D**) Both WT and Y98F HA can be assembled into nanoparticles of ferritin to display 8mers of the HA trimer^34^,^38^, that have overlapping elution profiles following size exclusion on a Superose 6 10/300 column.

HA from influenza A virus is a trimeric glycoprotein that binds cell surface sialyl-oligosaccharide with α2,6 (and to a lesser extent, α2,3) glycosidic linkages, through the receptor binding site, a conserved shallow pocket at the membrane-distal end of each protomer^30^,^31^,^32^,^33^,^34^. Structurally this interaction has been mapped extensively (Fig. 1) and within the RBS substitution of tyrosine for phenylalanine at position 98 (Y98F) prevents SA-binding^24^,^25^,^34^. Importantly this mutation does not disrupt the integrity of the RBS or the HA folding, leading to use of Y98F HA as a flow cytometry probe to identify antigen specific B cell responses^34^,^35^. We used this structurally described mutation as a tool to define whether SA-specific lectin activity activates B cell responses. To this end we generated recombinant versions of both wildtype (WT) and Y98F HA^34^,^35^ and confirmed their trimeric structures by size exclusion chromatography and conformational antibodies (Fig. 1A). Insertion of the Y98F mutation did not affect trimeric assembly, nor the binding of two conformational antibodies: CH65, which recognizes the RBS itself; and CR6261, specific for the functionally conserved HA-stem domain^36^ (Fig. 1B). CH65 possesses a unique CDRH3 that extends into the RBS pocket, making little contact to adjacent non-RBS structure^37^, making it an optimal tool to assess whether RBS-specific mutations disrupt the integrity of this protein domain. Consistent with this data, we previously showed by surface plasmon resonance that Y98F substitution, does not alter the association/dissociation kinetics and affinities of CH65, CR6261 or C05 (another RBS specific antibody) for this protein^34^. Attenuation of SA-dependent binding to the human B cell surface by the Y98F mutation was confirmed by flow cytometry (Fig. 1C). To increase the valency of this Tyr98-defined SA binding, the both WT and Y98F versions of the HA protomer can be attached to self-associating ferritin to produce nanoparticles displaying 8mers of the WT or Y98F HA trimer (Fig. 1D), as previously demonstrated^34^,^38^.

### The BCR is heavily sialylated, providing a substrate for SA-binding HA

**Figure 2:**
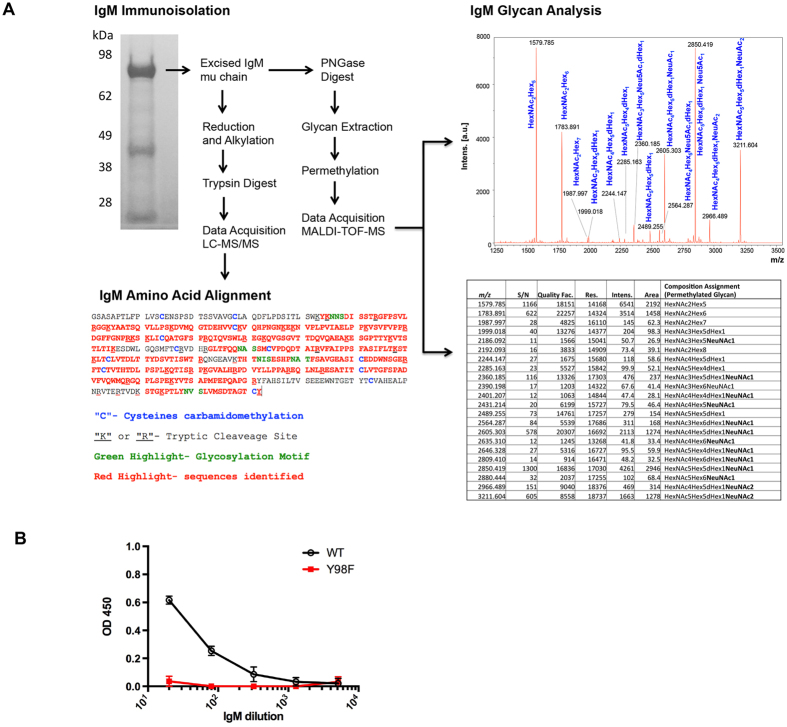
B cell IgM is heavily sialylated and binds to HA. (**A**) Secreted B cell IgM was immunoisolated (silver stain, upper left) and the mu chain gel band was excised for MS analysis. The IgM LC MS/MS and analysis first confirmed the IgM protein sequence (lower left) and MALDI-TOF-MS was used to analysis the glycans cleaved using PNGase. The spectra for this carbohydrate analysis is presented along with a summary table of the glycan structures. Abbreviations: Hex = Hexose; HexNAc = N-acetylhexosamine; dHex= deoxyhexose; NeuNAc = N-acetylneuraminic acid (=sialic acid; bolded in the summary table). (**B**) SA-dependent binding to secreted IgM was confirmed by the interaction between non-cognate IgM and WT HA but not Y98F HA. This binding was assessed by ELISA and is presented is the mean and sem of three independent binding replicates.

**Figure 3:**
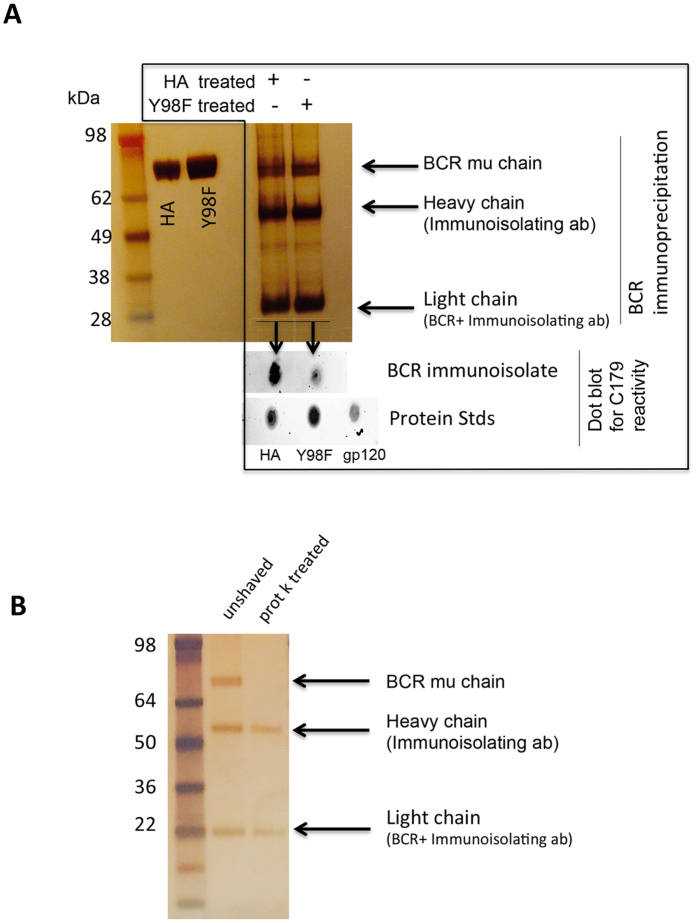
HA co-precipitates with BCR in a Tyr98 dependent manner. (**A**) B cells were exposed to WT or Y98F HA and the BCR was immunoisolated using an antibody specific for the IgM mu chain. The silver stained SDS PAGE of HA inputs and subsequent BCR immunoisolation is presented. To assay for the presence of HA, the immunoisolate was blotted for reactivity to C179, an antibody specific for the conserved stem region of HA^56^. (**B**) To confirm that the immunoisolation procedure was capturing cell surface bound BCR and not immature BCR within the cell, we also performed the procedure after surface B cell shaving with proteinase K^57^. The BCR was not isolated following surface shaving indicating that the procedure largely captures surface trafficked receptor.

Antigen lectin activity for SA has been previously implicated in B cell activation^21^, however a mechanism has not been assigned. The IgM BCR is one of the most abundant cell surface proteins known^39^, and we hypothesized that oligosaccarhide present on this receptor could provide a gateway for receptor stimulation. To verify that IgM is sialylated, we immunoisolated secreted IgM from Ramos B cells (a human B cell line^40^,^41^ that we subsequently modify to measure sequence-defined BCR responses to antigen, see below) and performed a glycopeptide analysis by mass spectrometry. We confirmed the both the protein sequence of our immunoisolate and identified multiple sialylated oligosaccharide sequences on it (Fig. 2A). Moreover we found that this SA signature facilitates Tyr98-dependent binding by HA when this IgM sequence is in soluble (Fig. 2B) and BCR formats (Fig. 3A,B).

### The naïve/germline BCR can be triggered by complementarity to antigen or lectin activity from the same antigen

**Figure 4:**
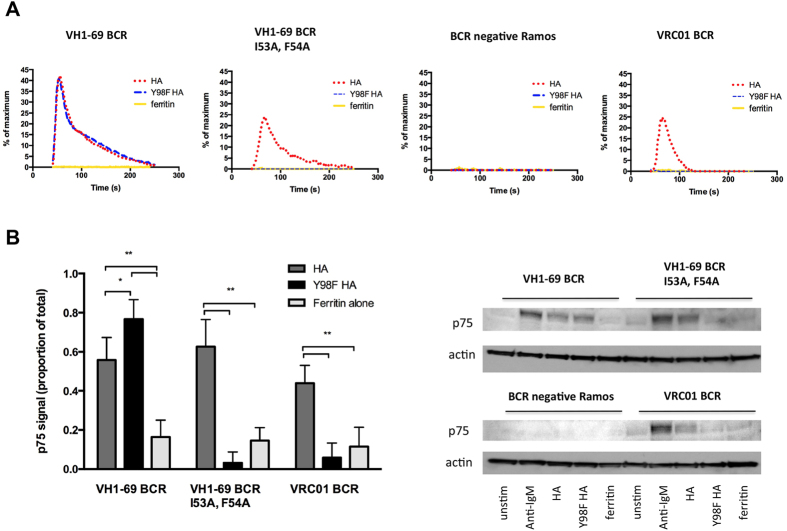
Reconstituted interactions between germline BCR and SA-binding HA reveal that the same BCR receptor can signal through both cognate and non-cognate/carbohydrate dependent interactions with antigen. Signaling as measured by Ca^2+^ flux (**A**) and tyrosine phosphorylation of p75^41^,^62^,^63^ (**B**) in response to ferritin nanoparticle arrayed HA trimer^38^ or Y98F HA trimer^34^ was assessed through Ramos B cells expressing: VH1-69 germline BCR specific for the HA stem; I53A, F54A VH1-69 germline BCR which prevents binding to the HA stem; Ramos B cell negative for surface BCR expression; or VRC01 BCR specific for the HIV envelope. Calcium flux was measured using the ratiometric calcium dye Fura Red, wherein flux in response to stimulation and is presented as the average of two independent runs, and for tyrosine phosphorylation, mean +/− sem of p75 signal (presented as a fraction of total signal seen with crosslinking with anti-IgM; uncropped immunoblots are provided in Supplementary Fig. 2) is presented for three independent experiments (**B**) (****P < 0.0001, ***P < 0.001, *P < 0.01). In all cases empty ferritin nanoparticle was used as an additional control.

To investigate the potential for cell surface SA to gate germline BCR antigen recognition and activation, we used a BCR signaling reporter system that we recently developed^40^ to reconstitute receptor output in response to WT versus Y98F HA. In this system we stably expressed defined BCR sequences of interest in a clone of Ramos B cells that we characterized as negative for endogenous surface IgM and then monitored calcium flux or tyrosine phosphorylation following antigen exposure^40^,^41^. In this case, we expressed germline-reverted IgM BCR for the *IGHV1–69*-derived antibody CR6261 (VH1-69 BCR) (Supplementary Fig. 1). CR6261 targets the HA stem region to confer broad neutralization activity against influenza virus^36^ and we have previously used its germline-reverted BCR to demonstrate how *IGHV1-69* gene usage centers antibody development on the stem epitope^40^,^41^. In parallel, we also introduced an I53A, F54A mutant of this BCR, which prevents its CDRH2-dependent recognition of the HA stem^40^,^41^. CDRH2-dependent reactivity to the HA stem was confirmed by the binding of Y98F HA trimer to VH1-69 BCR, but not VH1-69 I53A, F54A BCR (Supplementary Fig. 1). Furthermore, Y98F HA trimer containing the stem-blocking mutation I45R, T49R^40^,^41^ did not bind to VH1-69 BCR (Supplementary Fig. 1). Soluble HA trimers, however, produce very low levels of antigen-specific BCR signaling in this system, requiring a further arrayed presentation of antigen, which we supply through the generation of ferritin HA nanoparticles^40^,^41^ (Fig. 1D). In this context, we found that wildtype HA induced VH1-69 BCR signaling independently from the CDRH2 stem-binding paratope (Fig. 4A,B; Supplementary Figs 2 and 3). By contrast, Y98F HA elicited a signaling response through stem-reactive VH1-69 BCR, but not through its CDRH2 mutant I53A, F54A. Thus there were two modes by which HA induced signaling through the same germline BCR: a CDR-dependent mode and a Tyr98-dependent mode independent of cognate interactions. Non-cognate signaling was potent, occurring at nanomolar concentrations of HA (Supplementary Fig. 4) and was independent of CDR configuration as Tyr98-dependent signaling was also induced by Ramos B cells expressing BCR for a control HIV-1 antibody (Fig. 4A,B; Supplementary Figs 2 and 3). This antibody, VRC01, has broad neutralization activity against HIV-1 and does not recognize influenza HA^42^. Both cognate and non-cognate modes of receptor signaling were dependent on the presence of BCR in the membrane as neither calcium flux nor tyrosine phosphorylation were seen in IgM negative B cells following exposure to HA (Fig. 4A,B; Supplementary Figs 2 and 3). Our data are therefore consistent with a model wherein HA, being at minimum trimeric for sialyl-oligosaccharide lectin activity, is able to ligate the BCR and/or BCR associated components leading receptor activation.

### SA-binding activity activates antigen naïve B cells from peripheral blood

**Figure 5:**
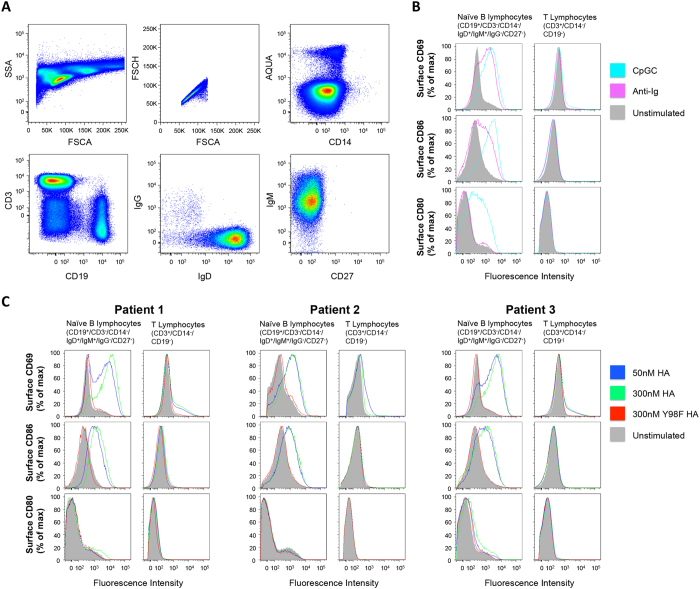
Recombinant influenza HA activates primary B cells in the absence of pre-existing antigen specificity. Freshly purified PBMCs were purified and incubated with B cell stimulators, nanomolar levels of HA or its sialic acid binding mutant Y98F HA and lymphocyte activation was evaluated by surface expression of CD69, CD86 and CD80 on naïve B cells or T cells. (**A**) Flow cytometry scheme: naïve B cells were defined as CD19^+^/CD3^−^/CD14^−^/IgD^+^/IgM^+^/IgG^−^/CD27^−^; and T cells in same preparation were defined as CD3^+^/CD14^−^/CD19^−^. (**B**) Histograms for B cell and T cell surface levels of CD69, CD86 and CD80 following incubation with known B cell stimulators (Ig or CpGC). (**C**) Histograms for B cell and T cell surface levels of CD69, CD86 and CD80 following incubation with 50 nM HA, 300 nM HA or 300 nM Y98F HA. Presented are the results of stimulating PBMC from three different individuals.

To test whether germline BCR signaling through non-cognate SA interactions could actually stimulate naïve B cell activation, we next evaluated primary B cell responses to lectin activity for SA. We observed a dramatic upregulation of the lymphocyte activation markers CD69 and CD86 on naïve B cells (CD19^+^/IgD^+^/IgM^+^/IgG^−^/CD27^−^ lymphocytes) after incubating human PBMC with WT but not Y98F HA (Fig. 5A–C). The lymphocyte activation marker CD80 was unaffected by HA exposure, a property also seen for BCR stimulation by anti-Ig crosslinking (Fig. 5B,C). This contrasted with B cell activation by bacterial CpG oligodeoxynucleotide (Fig. 5B,C), a TLR9 ligand that stimulates through a BCR-independent pathway^43^. As with reconstituted BCR signaling, Tyr98-sensitive activation was potent, with an onset occurring at nanomolar concentrations of HA and was most dramatic for B cells since T cells within the same PBMC preparation showed limited CD69 and CD86 upregulation (Fig. 5C). Notably, naïve B cells did not require further multimerization of HA on a nanoparticle support, as was necessary for reconstituted BCR signaling. To explore the molecular basis for this difference we measured the number of sequenced-defined BCRs ectopically expressed at the surface of our reporter cell line. Resting B cells display ~100000–200000 BCR copies at the surface^39^ and using flow based receptor copy measurements^44^ we obtained value of 11599 ± 676 surface receptors in our reconstituted BCR signaling system (Supplementary Fig. 5), a logfold reduction in surface receptor valency that is consistent with the need to enhance antigen valency to facilitate receptor activation.

As additional readout for primary B lymphocyte activation, we also tested whether Tyr98-dependent activation could drive primary B cell differentiation *in vitro*. Activation of purified naive CD19^+^/IgM^+^ B cells through BCR crosslinking induces surface expression of CD27, a memory marker indicating differentiation away from an antigen-naïve state^45^. Accordingly, we purified these cells from peripheral blood and incubated them with nanomolar concentrations of antigen (Supplementary Fig. 6). Cells treated with WT but not Y98F HA reproducibly elicited increases in surface CD27 levels, comparable to what was observed with crosslinking with anti-Ig (Supplementary Fig. 6).

## Discussion

Ligation of surface SA as a gateway to antigen naïve BCR activation offers a previously unrecognized mode of initiating B cell responses to antigen and demonstrates that B cells have an activation modality akin to the well-described effects of lectins on T cells^11^,^12^,^13^,^14^,^15^. Presented is a biochemical and cellular capability that we have explored using a structurally defined viral protein- host carbohydrate interaction^24^,^25^,^26^,^27^ and a new platform for reconstituting germline BCR signaling activity. Our data indicate that in the presence of antigen affinity for SA, the BCR signal can be formed through either cognate or carbohydrate-dependent non-cognate input, and that the latter induces pan B cell activation. Pan B cell stimulation or superantigen activity is a property which some microbial products employ to dampen adaptive immune responses^28^,^29^ and our reconstitution experiments indicate that SA-binding through a viral lectin provides a similar immune activation modality.

While the precise mechanism of receptor activation remains debated, the BCR has long been known to be triggered through surface clustering interactions seeded by multivalent antigen^5^,^6^,^46^. Our reconstituted germline receptor activity measurements revealed that: 1) HA binds to B cell IgM SA directly (Figs 2 and 3); 2) Tyr98-dependent binding to B cell SA triggers BCR activation (Fig. 4); and 3) Tyr98-dependent activation was reliant on the presence of the BCR itself (Fig. 4). Importantly, this demonstrated two modes of activating the same germline antigen receptor: triggering that proceeded through CDR-dependent complementarity and triggering that was dependent on antigen affinity for cell surface carbohydrate. The SA-dependent arm of BCR signaling occurred at nanomolar concentrations of HA, indicating that glycan-dependent interactions with antigen form a potent stimulator of naïve BCR receptor activity. Glycan-dependent BCR signaling was further confirmed through Tyr98-dependent activation of VRC01 BCR, an antibody sequence that does not bind HA. Both modes of activation were dependent on the presence of BCR in the membrane indicating that SA-dependent triggering involved either direct ligation of the BCR itself and/or adjacent SA displaying glycoproteins. The presence of BCR sialylation was confirmed by glycoproteomic analysis of B cell IgM, consistent with previous glycoform analyses^47^ and the effect of treatment of this receptor with sialidase^48^. Moreover, direct Tyr98-dependent ligation of the IgM BCR indicated that affinity for receptor oligosaccharide likely seeds its clustering and activation. We do not preclude ligation of associating BCR components as effectors in this activation process; and in either case the key functional consequence is the same – a previously unrecognized mode of BCR activation produced through non-cognate/glycan-dependent interactions with antigen.

SA-dependent simulation of reconstituted germline BCR receptor activity predicted that the antigen naïve B cell could be activated by affinity for SA, independent of cognicity for antigen. Crosslinking of Ig receptors in the absence of complementarity is a strategy employed by microbial superantigens to circumvent the adaptive immune response^28^,^29^. Here activation of the antigen recognition machinery in the absence of antibody-complementarity for antigen can compete with and depress the capacity to generate antigen-specific responses^28^,^29^. To test for B cell superantigen activity, we evaluated HA-responses from antigen naïve primary B cells isolated from peripheral blood. Akin to our BCR readout, we demonstrated Tyr98-dependent stimulation of this lymphocyte subset, with pan-activation by SA-binding HA, underscoring the non-cognate feature of this signaling process (Fig. 5). Indeed, an initial rise in B cell surface CD69 and CD86, but not CD80 is a hallmark of a BCR-centered superantigen response^29^, and in contrast to its upregulation upon induction TLR signaling, no change in CD80 was observed following BCR ligation by either anti-Ig or SA binding (Fig. 5). CD69 and CD86 were not elevated in response to cognate interactions (i.e. no HA-specific B cell responses were detected) despite the fact that these are well-characterized markers for early B cell activation following immunization^49^,^50^. This highlights the difference in dimensionality through which cognate and non-cognate B cell specificities operate. Germline antibody recombination can produce strong antigen-specific binding partners, such as germline VH1–69 BCRs which favor cognate interactions with the HA stem^40^,^41^, however the number of BCRs having biochemical specificity for antigen will be low relative to the diversity of the repertoire^1^,^2^,^3^,^4^. By contrast, pan-activation relies on a set BCR affinity that is not supported by a receptor diversification and selection process. While this means potentially less optimized germline specificity, superantigen activity is compensated by virtue of its global reach. Modulation of BCR signaling has previously been reported by lateral input from SA-bearing Siglecs, namely CD22^18^,^19^. On resting cells, CD22 interacts with α2,6-linked sialic acid (SA) moieties in *cis*-forming homo-oligomers. Disruption of these interactions via SA-dependent antigen contact in *trans* can result in increased association of CD22 with the BCR and subsequent inhibition of signaling and increased antigenic tolerance^18^,^19^. However in our case, HA mediated a SA-dependent *increase* in B cell activation, a finding that does not support *trans* interference with CD22 homo-oligomers to dampen BCR signaling. As further confirmation of an activating process, we found that both crosslinking through anti-Ig and SA binding by HA lead to upregulation of the memory marker CD27 in our B cell differentiation assay, indicating that akin to crosslinking through anti-Ig, ligation of BCR SA can induce differentiation in the absence of conventional antigen complementarity and forms the basis for a carbohydrate-dependent superantigen activity.

Biology is replete with identifying new activation pathways and substrate specificities through enzyme/receptor reconstitution approaches^51^,^52^. We have employed new methodology for reconstituting BCR signaling along with a structurally characterized glycan-protein interaction to demonstrate that the human germline BCR can be induced to signal via direct ligation of surface carbohydrate. This receptor activity was independent from BCR triggering through antigen complementarity, and manifested as a pan stimulation of primary naïve B cells isolated from human blood. Pan B cell activation is a known superantigen activity used by some pathogens to avoid immune surveillance as it reduces the possibility of CDR-antigen pairing and the generation of antigen-specific immune responses^28^,^29^. Given this now additional carbohydrate-based mode of pan-B cell signaling, it will be of interest to define how it operates during the generation of humoral immunity, particularly in response to human SA-binding pathogens, for which there are many^22^,^23^.

## Methods

### Recombinant proteins

All proteins were expressed in 293F cells using pVRC8400, a plasmid containing the CMV IE Enhancer/Promoter, HTLV-1 R Region and Splice Donor site, and the CMV IE Splice Acceptor site upstream of the open reading frame^42^. The soluble trimeric or ferritin nanoparticle formats of the wildtype or Y98F HA trimer from A/New Caledonia/20/1999 (H1 1999 NC) were as previously described^34^,^38^,^40^. The monoclonal antibodies CR6261^36^, CH65^37^ and VRC01^42^ were also expressed and purified described previously^31^,^32^.

### Enzyme-linked immunosorbant assay (ELISA)

WT HA trimer or Y98F HA trimer were coated overnight onto MaxSorp plates (Nunc) at 2 μg/ml. The plates were then incubated (1 h) with serial dilutions of HA-RBS reactive CH65, HA-stem reactive CR6261 or HIV envelope-specific VRC01 as an isotype control. The wells were then washed, incubated with a 1/5000 dilution of sheep anti-mouse HRP conjugated IgG or sheep anti-human HRP conjugated IgG (GE Healthcare), washed again and developed using one-step TMB substrate (Dako). This reaction was stopped by the addition of 2M sulphuric acid and the plates were then read at 450 nm using a Spectomax Plus (Molecular Devices).

### Glycan Analysis of IgM

Ramos cells were initially maintained in cRPMI supplemented with 15% FBS, penicillin/streptavidin, and 1X L-glutamine. To isolate secreted IgM from this cell line, cells were then incubated in serum-free RPMI for 72 hours prior to immunoisolation^53^. Twenty million Ramos were then pelleted cells at 1250 rpm for 10 min. Supernatant was collected and was concentrated to 250 μl (Amicon 30 K concentrator). Concentrated supernatant was then applied onto 100 μl of PBS-equilibrated capture Select IgM Affinity matrix beads (Life Technologies) beads and incubated rotating at 4 °C for 4 h. The supernatant was removed and beads were washed 2 times with ice-cold PBS. Beads were pelleted via centrifugation at 1500 rpm for 5 min. Residual buffer was then removed and beads were resuspended in 50 μl SDS reducing buffer. Samples were boiled for 5 min at 100 °C. The supernatant containing the bound protein was then obtained after centrifugation at 1500 rpm for 5 min. Samples were then separated by SDS PAGE and stained with Gelcode Blue (Pierce). The IgM mu chain (~70 kDa) protein band was excised and cut into 1 mm^3^ for processing. It was then washed with 25 mM ammonium bicarbonate in 50% acetonitrile until completely destained. The gel piece was dehydrated with 100% acetonitrile and dried in SpeedVac prior to enzymatic processing. The dried gel piece was then treated with PNGase F (750units in 250 μL 1x GlycoBuffer) for 18 hrs at 37 °C. Glycans were extracted by removing the supernatant which already may have contained some oligosaccharides and combining with two 500 μL water exchanges with mixing and two 400 μL water exchanges with sonication for 30 mins each. Glycan extract was dried to completion in a SpeedVac. Glycans were resuspended in 50 μL 20% acetonitrile prior transferring to a small glass vial for permethylation reaction. Released glycans were permethylated according to established protocols^54^,^55^, as follows. The dried samples were dissolved in 1 μL water and 65 μL DMSO, followed by addition of 100 μL NaOH beads (Sigma) in DMSO. To initiate the permethylation reaction, 16 μL iodomethane was added and the samples were mixed gently for 1 hour in the dark. Addition of iodomethane was repeated twice more within the hour, followed by liquid phase extraction using chloroform and water, repeated 4 times. Glycans in the chloroform phase were dried in a Speed vac. Dried samples were resuspended in 50% methanol with 1 mM sodium acetate and spotted onto a MALDI plate. An equal volume of 2, 5-dihydroxybenzoic acid solution (20 mg/mL in 50% acetonitrile) was spotted on top of the glycan sample and dried. MALDI-TOF MS of the permethylated glycans was performed on a Bruker Ultraflextreme TOF/TOF using 60% laser and 500–1500 shots.

### Proteome Analysis of IgM

Following glycan extraction, the IgM gelband was dehydrated with 100% acetonitrile and reduced with 10 mM DTT in 25 mM ammonium bicarbonate for 30 mins at 37 °C. After cooling to room temperature and removal of reduction solution, the gelband was alkylated with 40 mM iodoacetamide in 25 mM ammonium bicarbonate for 45 min in the dark at room temperature. The gel piece was then washed with water, dehydrated with 100% acetonitrile and dried in a SpeedVac prior to trypsin digestion. 15 ng/μL of trypsin in 25 mM ammonium bicarbonate was added to cover the gel piece for overnight digestion at 37 °C. Peptides were extracted by removing the supernatant and combining it with three sequential extractions with 50% acetonitrile and 5% formic acid for 20 mins while mixing. The sample was chromatographically separated using Proxeon Easy NanoLC 1000 (Thermo Scientific) fitted with a PicoFrit 75-μm capillary (New Objective) self-packed with 20 cm of C_18_–AQ ReproSil-Pur beads (1.9 μm, 200 Å, Dr. Maisch GmBH) at 50 °C using a column heater. Samples were reconstituted and loaded in 3%ACN, 0.1%FA at a flow rate of 500 nl/min. Peptides were eluted at a flow rate of 200 nL/min using a linear gradient from 6–30% Buffer B over 84 min, 30–60% Buffer B over 9 min, a quick ramp up to 90% Buffer B over 1 min and held for 5 min followed by a quick ramp down to 50% Buffer B over 1 min and held at 50% for an additional 9 min (Buffer A: 3%ACN and 0.1%FA; Buffer B: 90%ACN and 0.1% FA). During data acquisition, eluted peptides were introduced into a Q-Exactive mass spectrometer (Thermo Scientific) via nanoelectrospray (2.15 kV). A full-scan MS was acquired at a resolution of 70,000 from 300 to 1800 m/z (AGC target 3e6, 5 ms Max IT). Each full scan was followed by top 12 MS2 scans at a resolution 17,500 (isolation width 2.5 m/z, ACG Target 5e4, 120 ms Max IT). The HCD collision energy was set to 25, the dynamic exclusion time was set to 20 s and the peptide match and isotope exclusion settings were enabled.

### MS analysis

MS data were processed and interpreted using the Spectrum Mill software package v4.1 beta (Agilent Technologies). MS/MS spectra acquired on the same precursor (m/z 1) with 60 s were merged and searched against a Uniprot database containing human sequences including isoforms and excluding fragments with a set of common laboratory contaminant proteins appended. The enzyme specificity was set to trypsin (cleavage at KP or RP allowed) and the maximum number of miscleavages was set to 2. The minimum matched peak intensity was set to 30% with +/−20 ppm precursor mass tolerance and fragment mass tolerance. Carbamidomethylation of cysteine was searched as a fixed modification. Pyroglutamic acid (N-term), oxidation of methionine and deamidation of asparagine were searched as variable modifications (precursor MH+ mass shift range −18 to 64Da).

### IgM-HA binding

Two hundred nanogram of Y98F or WT HA was coated onto MaxSorp plates for ELISA, as described previously. Secreted IgM from wildtype Ramos was prepared as described for the glycan analysis, however, in this case, the serum-free IgM supernatant concentrate was added to the microtiter plates in serial dilution and the ELISA proceeded as described above, except that the secondary antibody was an anti-human IgM-HRP (Novus, NBP1–75035) used at 1/5000.

### IgM BCR Immunoprecipitation

Twenty million Ramos cells were washed with ice cold PBS and incubated with 0.5 μM WT HA or Y98F HA for one hour at 4 °C. The cells were then pelleted and washed five times with ice cold PBS. The cells were then resuspended in 150 μl ice cold HBS lysis buffer (50 mM HEPES, 150 mM NaCl, 1% TX-100, 2 mM EDTA, 1X Protease inhibitor cocktail, Roche, pH 7.4) and incubated for 30 min at 4 °C. During this time, anti-human IgM beads (μ-chain specific, Sigma A9935) were equilibrated in HBS lysis buffer at 4 °C. The cell lysis solution was then centrifuged at 1500 rpm for 5 min and the supernatant was added to 100 μl of equilibrated anti-human IgM beads. The mixture was then incubated on a rotator at 4 °C for 4 hr. The beads were then washed twice with HBS lysis buffer, residual buffer was removed and beads were resuspended in 50 μl SDS reducing buffer. Membrane bound BCRs were eluted by boiling the samples at 100 °C for 5 min and centrifuging at 1500 rpm for 10 min. Supernatant, which contained the eluted protein, was carefully pipetted off and analyzed by SDS page or dot blotting using the HA stem specific antibody C179^56^. To confirm that this procedure isolated surface BCR and not immature BCR still inside the cell, the pelleted B cells were subjected to surface shaving by proteinase K^57^ (2 h 37 °C in PBS ± 10 mg/ml proteinase K) and the immunoisolation procedure was then repeated.

### Reconstituted BCR signaling

BCRs for germline CR6261, germline CR6261 I53A, F54A, and VRC01 were stably expressed in a previously described surface IgM negative clone of Ramos B lymphocytes^41^ by means of a lentiviral vector. The lentiviral vectors were produced in 293T cells following transfection of the following plasmids: pFEEKW (a lenti viral expression vector containing the membrane bound IgM or antibody light chain transgenes of interest^58^); pVSVG (vesicular stomatitis virus glycoprotein for envelope pseudotyping^59^); and p∆R8.2 (a packaging vector^60^). BCR positive cells [defined as staining positive for both light chain (mouse PE-anti-human lambda chain, BD Biosciences) and IgM (APC-anti-human IgM, BioLegend)] were then sorted by flow cytometry (FACSAria II, BD Immunocytometry Systems) and enriched (Supplementary Fig. 1). Cells were evaluated for triggering when BCR reached >90% expression. All BCR sequences were enriched using an identical gating strategy. To define the number of BCR receptor copies that our lentivirus expression system produces, we employed the immunocytochemical flow receptor quantification developed by Lopez *et al*.^44^. A standard curve was generated from MFI against beads with defined molecules of equivalent soluble fluorochrome (Bangs Laboratories Inc). For a monomeric probe to count BCR, the gp120 core RSC3^42^ was labeled with an Alexa 488 labeling kit according to the manufacturers instructions (ThermoFisher Scientific). After a 1:1 protein to dye ratio was verified through absorbance measurements, the probe was applied to VRC01 BCR cells at saturating levels and the MFI was used to calculate the BCR copy number from the standard curve.

To measure the kinetics of signaling, calcium flux in response to BCR stimulation was measured by flow cytometry (LSR II, BD Immunocytometry Systems) as the ratio of the Ca^2+^ bound/unbound states of the dye Fura Red^61^. Here 1 × 10^6^ cells were exposed to 2.5 μM HA or 2.5 μM Y98F HA [a concentration previously established to induce germline VH1-69 BCR signaling with HA ferritin (the HA trimer alone is a weak inducer of signaling in this system)^40^,^41^; however, the onset of sialic acid-dependent signaling was also measured at 0.1 μM HA (Supplementary Fig. 4)] or 0.5 μg/μl mouse anti-human IgM F(ab’)_2_ (Southern Biotech). Empty ferritin nanoparticle^38^ was used at the same concentration as a negative control. Ratiometric measures for individual cells were averaged, smoothened (Kinetic analysis, Flow Jo software) and normalized to total Ca^2+^ flux as measured by exposure of cells to 10 μg/ml ionomycin. For endpoint measurements, 4 × 10^6^ cells were exposed to anti-human IgM F(ab’)_2_ or HA ferritin nanoparticles as above. After 15 min exposure at room temperature, cells were placed at 4 °C, washed two times with PBS and then lysed for 10 min in lysis buffer (Cell Signaling Technology) supplemented with protease inhibitors (Complete Protease Inhibitor Cocktail, Roche Applied Science). Following SDS PAGE, BCR activation was assessed by Western analysis of the cell lysate: 4G10 pY (Millipore) reactivity to p75 as described^41^,^62^,^63^. Total phosphotyrosine intensity was measured by densitometry [Image Processing in Java (Image J) Software + curve area density calculation in Microsoft Excel]. For each BCR, phosphotyrosine intensity was standardized to the level of the anti-human IgM F(ab’)_2_.

### Antigen naïve B cell stimulation PBMCs *ex vivo*

Leukopacs were obtained from the MGH blood bank. PBMCs were isolated^64^ and resupended in RPMI containing 10% FBS (R10) + benzonase [50 U/ml, Millipore], pelleted (1650 rpm for 5 min) and then divided into 6 groups (each containing 10 million cells) for B cell stimulation: unstimulated control; Anti-Ig control [5 μg/ml, F(ab’)_2_ Fragment Goat Anti-human IgA + IgG + IgM (Jackson)]; CpG-C control [2 μg/ml CpG-C (Invitrogen) +1 ug/ml Il-2 (Miltenyl Biotech)]; 50 nM WT HA; 300 nM WT HA; and 300 nM Y98F HA. In these experiments HA ferritin nanoparticle was not required for stimulation, so we proceeded with trimeric HA versions only. Activation took place at 37 °C over a 20 h period. Following this time, PMBC were resuspended in 50 ul of PBS with Aqua Live/Dead amine-reactive dye (Invitrogen) (0.025 mg/ml for 2 minutes), quenched by addition of 50 ul 1% FBS in PBS, and then stained with the addition of a 50 ul 1X cocktail of B cell-, monocyte- and T cell-specific flow cytometry antibodies: CD19-ECD (Beckman Coulter); CD14-QD800, CD27-QD605 (Invitrogen); CD3-Cy7APC IgM-Cy5.5-PerCP, IgG-BV421, IgD-Cy7PE, CD69-FITC, CD80-PE (BD Pharmingen); CD86-BV650 (Biolegend). Staining was for 1 h at 4 °C. The cells were then washed twice resuspended in 400 μl of PBS and then analyzed by flow cytometry. Between one and two million events were collected on an LSR II instrument (BD Immunocytometry Systems) configured to detect 18 fluorochromes. Compensation for this staining panel was achieved using anti-mouse Ig kappa Compbeads (BD Biosciences) and anti-mouse Ig lambda Compbeads (Spherotech). Analysis was performed using FlowJo software version 9.5.2 (TreeStar).

### Stimulation of Purified naïve B cells

Naïve B cell isolation kit II human (Miltenyi Biotec) was used to isolate naïve B cells from freshly isolated PBMCs according to the manufacturer’s instructions. One hundred thousand naïve B cells were stimulated with WT HA, or Y98F HA, each at 50 nM in RPMI containing 10% FBS. Control cells were stimulated with Anti-Ig [5 μg/ml, F(ab’)_2_ Fragment Goat Anti-human IgA + IgG + IgM (Jackson)]. After four days, the cells were washed in PBS and resuspended in 50 ul of PBS with Aqua Live/Dead amine-reactive dye (Invitrogen) (0.025 mg/ml for 2 minutes), quenched by addition of 50 ul 1% FBS in PBS, and then stained with a 1X cocktail of following antibodies: CD19 ECD (Beckman), CD27 QD605 (Invitrogen), IgM Cy5PerCP (BD Pharm) for 1 hour at 4 °C. The cells were then washed twice resuspended in 400 μl of PBS and then analyzed by flow cytometry.

### Statistics

Data is presented as mean +/− SEM. For multiple comparisons, statistical significance was assessed by ANOVA with Tukey’s test. All analyses were performed using Graphpad Prism version 5.0 (GraphPad Software Inc.).

## Additional Information

**How to cite this article**: Villar, R. F. *et al*. Reconstituted B cell receptor signaling reveals carbohydrate-dependent mode of activation. *Sci. Rep.* **6**, 36298; doi: 10.1038/srep36298 (2016).

**Publisher’s note:** Springer Nature remains neutral with regard to jurisdictional claims in published maps and institutional affiliations.

## Supplementary Material

Supplementary Information
