# Supplementary material for: Reconstituted B cell receptor signaling reveals carbohydrate-dependent mode of activation
Source: Sci Rep. 2016 Oct 31;6:36298. doi: 10.1038/srep36298 (PMC5087089; doi:10.1038/srep36298)
Supplement: Supplementary Information [file srep36298-s1.pdf]

## Supplementary Information

# Reconstituted B cell receptor signaling reveals carbohydrate-dependent mode of activation

Rina F. Villar, Jinal Patel, Grant C. Weaver, Masaru Kanekiyo, Adam K. Wheatley, Hadi M Yassine, Catherine E. Costello, Kevin B. Chandler, Patrick. M. McTamney, Gary J Nabel, Adrian B McDermott, John R Mascola, Steven A. Carr and Daniel Lingwood

Includes: Supplementary Figure Legends

**Supplementary Figure Legends**

Supplementary Figure 1. Antigenicity in the BCR reporter cell line. Heavy chain and light chain lenti viral vectors were used to express the following BCRs in Ramos devoid of endogenous surface membrane IgM: germline CR6261 (=VH1-69 BCR) as wildtype or with its HA stem binding mutation I53A/F54A; and VRC01. Presented is BCR antigenicity for HA. Wildtype HA binding is in red and ΔHA binding is in blue (ΔHA = introduction of I45R and T49R mutations into HA2 to prevent stem binding). The grey in these panels represents binding to VRC01 BCR-displaying Ramos. The purple in the bottom panels illustrate the surface trafficking of wildtype and mutant CR6261 BCR as measured by staining with PE-labeled anti-lambda IgG. The grey in these panels is VRC01 BCR displaying Ramos (VRC01 uses a kappa chain).

Supplementary Figure 2. Uncropped BCR immunoblots (see Fig. 4B) using 4G10 with low exposure time (A) and high exposure time (B). BCR signaling was in response to ferritin nanoparticle arrayed HA trimer or Y98F HA trimer was assessed through Ramos B cells expressing cells expressing: VH1-69 germline BCR specific for the HA stem; I53A, F54A VH1-69 germline BCR which prevents binding to the HA stem; Ramos B cell negative for surface BCR expression; or VRC01 BCR specific for the HIV envelope. Empty ferritin nanoparticle was used as an additional control. The position of p75 induction in the 4G10 immunoblot upon BCR crosslinking is denoted on the far left.

Supplementary Figure 3. Uncropped BCR immune blots (see Fig. 4B; Supplementary Fig. 2) using anti-actin with low exposure time (A) and high exposure time (B).

Supplementary Figure 4. Titration of Tyr98-dependent activation as triggered through I53A/F54A germline CR6261 BCR in response to HA ferritin nanoparticles (0.5-0.1μM), Y98F HA ferritin nanoparticles (2.5μM) and empty ferritin nanoparticles (2.5μM). BCR signal in response to IgM crosslinking is shown on the left.

Supplementary Figure 5. BCR copy number in following lentiviral expression in IgM negative Ramos B cells. (A) Alexa 488 beads with a known number of molecules of equivalent soluble fluorochromes (MESF) were used to create a standard curve with MFI. (B) RSC3, a monomeric gp120 core protein was labeled with Alexa-488 (protein to dye label =1:1) and applied to IgM negative or VRC01 IgM BCR expressing cells at a saturating level. The corresponding MESF value was calculated and the receptor copy number was obtained.

Supplementary Figure 6. SA binding proteins induce CD27 expression on $CD19^+$/$IgM^+$ antigen naïve B cells. (A) $CD19^+$/$IgM^+$ B cells were purified from freshly isolated PBMC using the Naïve B cell isolation Kit II (Miltenyi Biotec) and (B) treated with anti-Ig, 50 nM WT HA, or 50 nM Y98F HA for four days. Acquisition of the memory marker CD27 was monitored at the end of this period. Presented are the corresponding

histograms for CD27 surface expression from $CD19^{+}/IgM^{+}$ B cells purified from three different individuals.

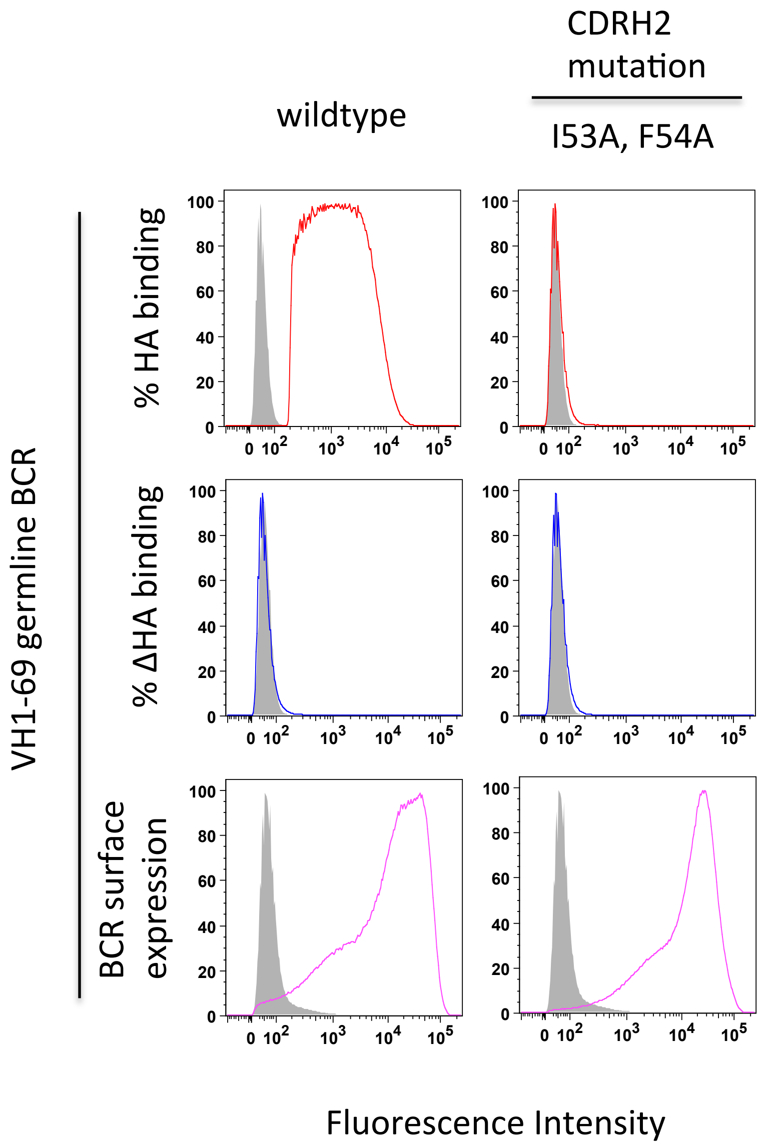


Supplementary Figure 1

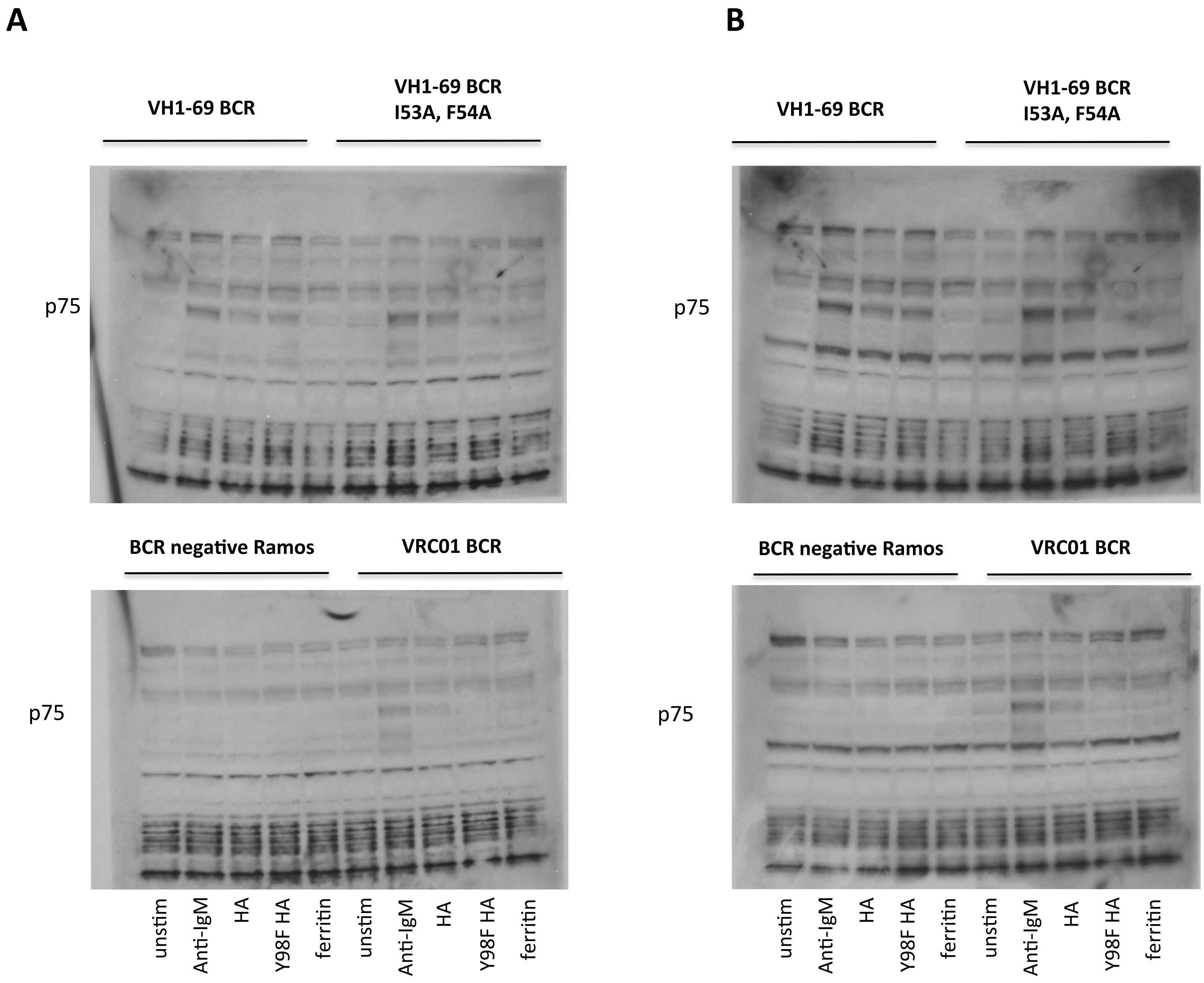


Supplementary Figure 2

**A**

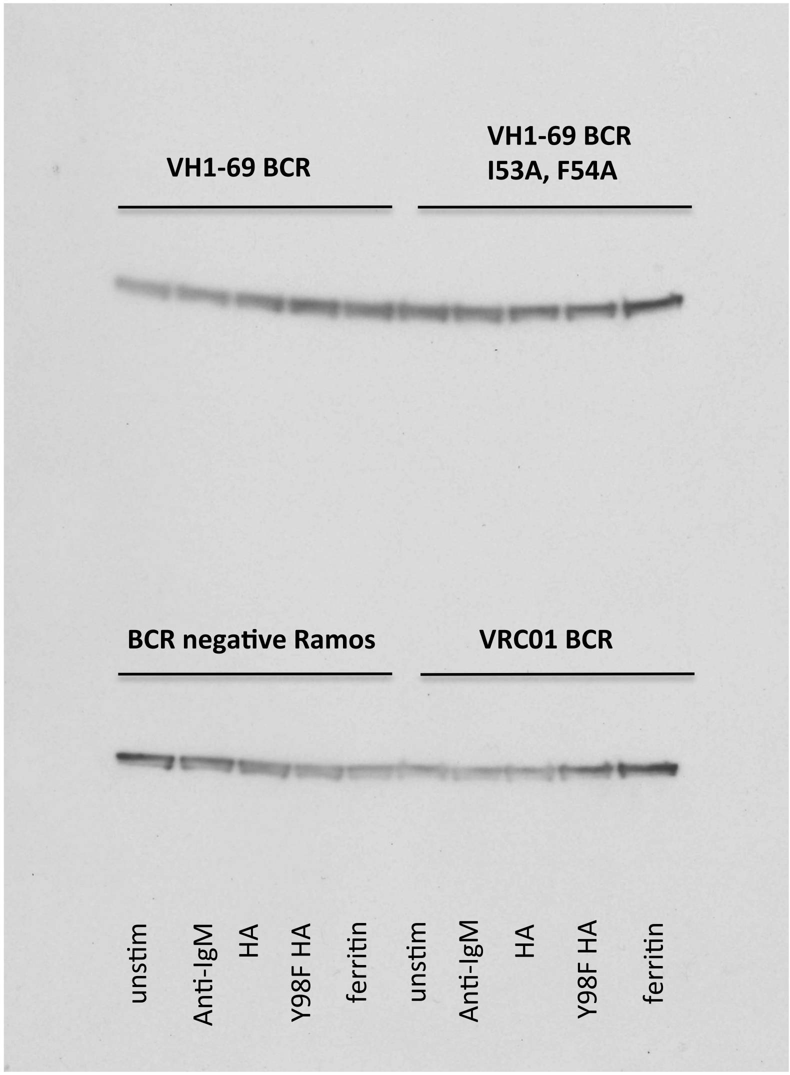


**B**

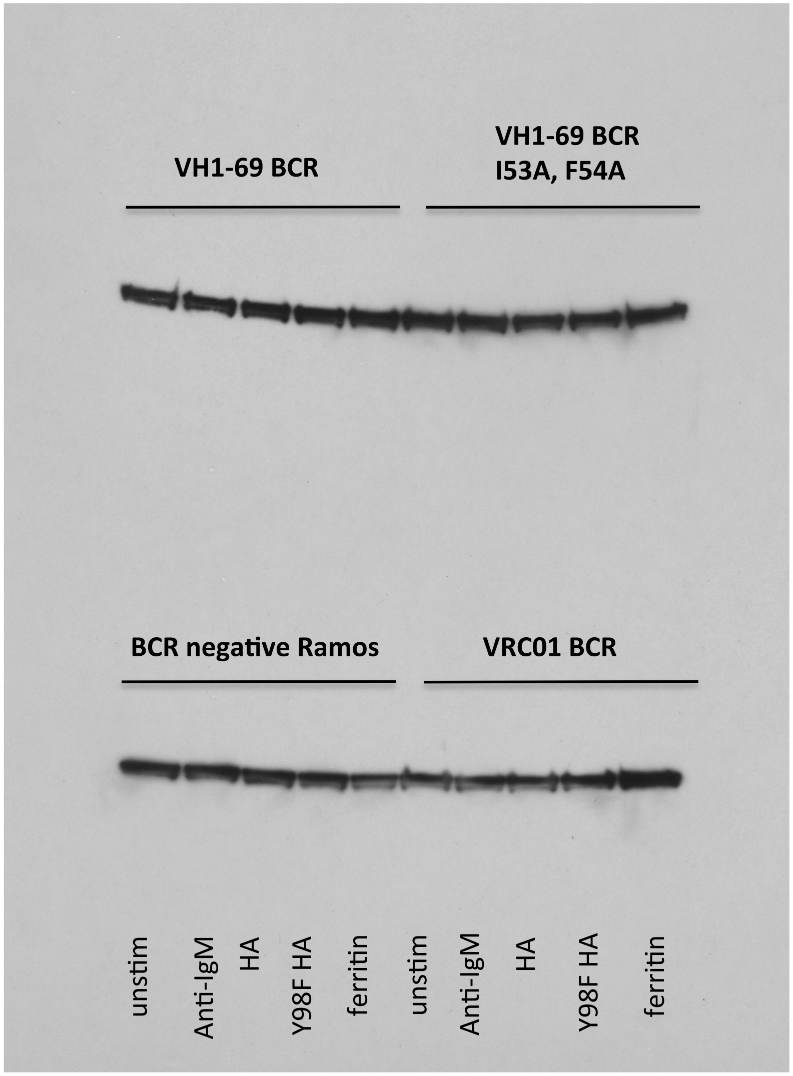


Supplementary Figure 3

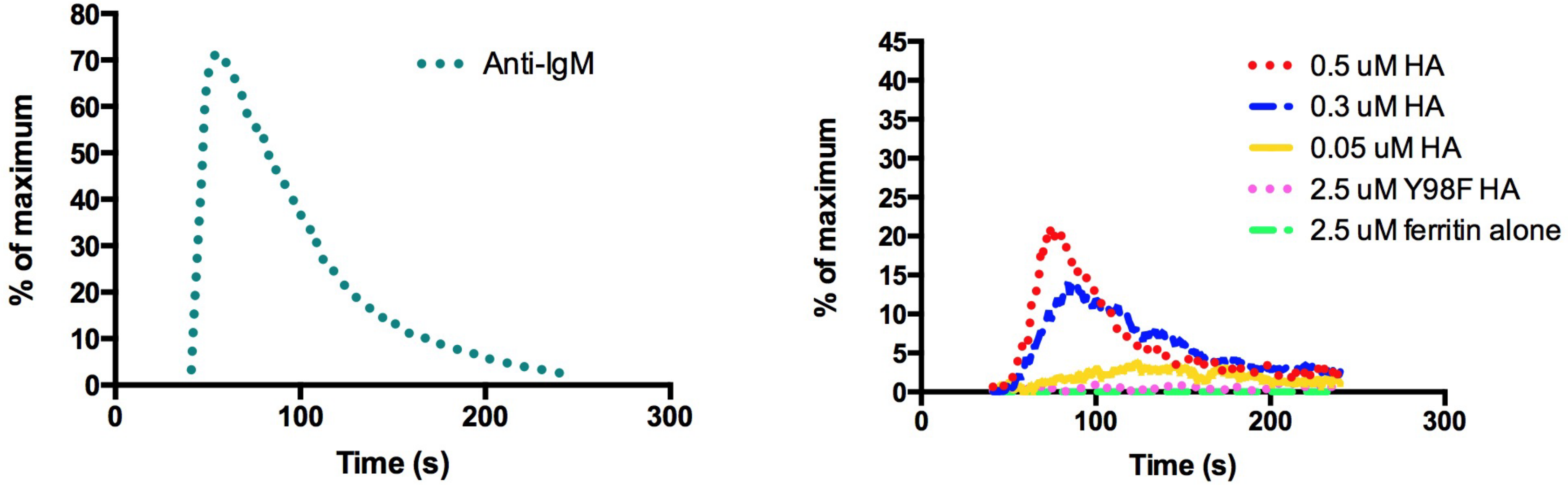


Supplementary Figure 4

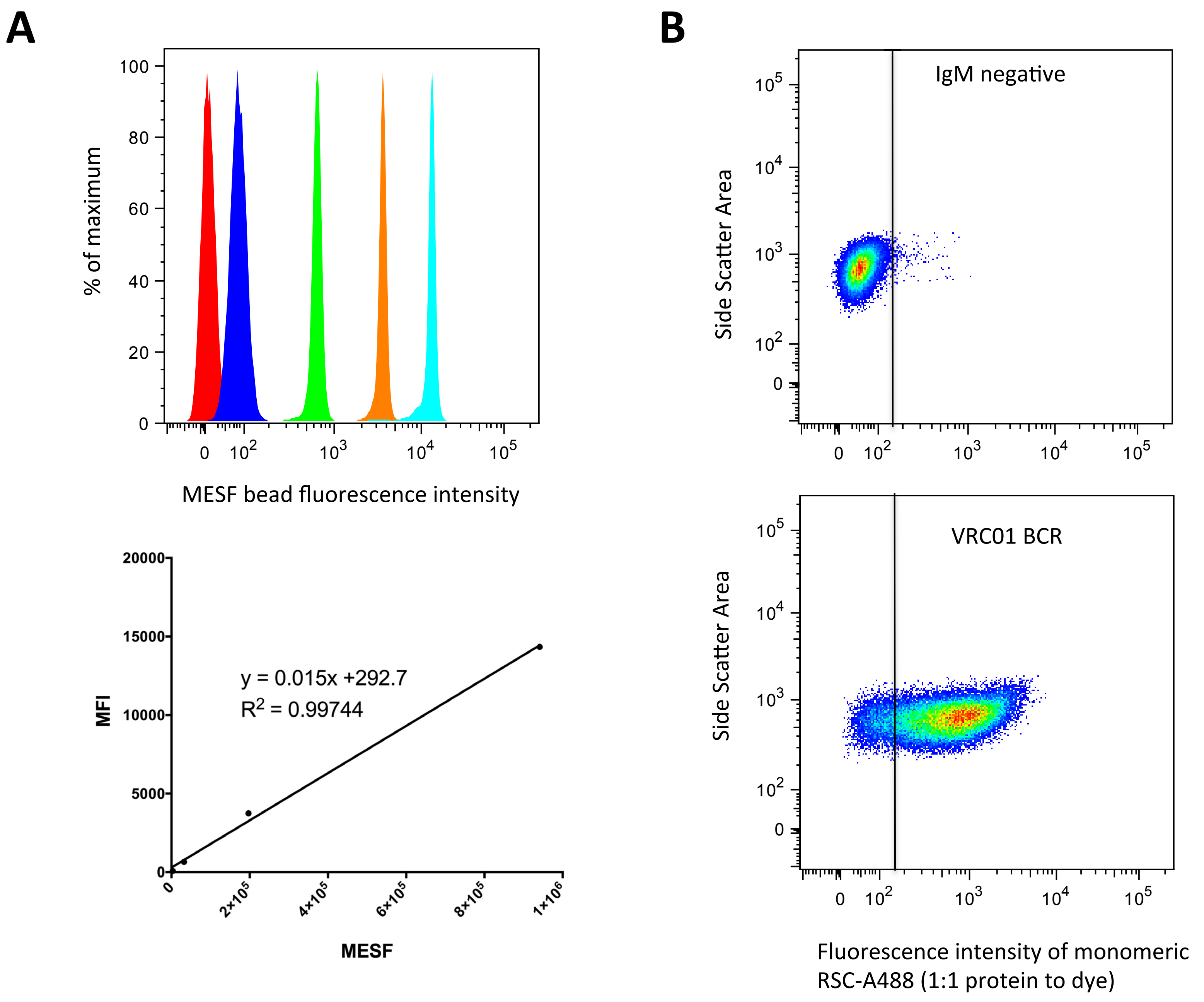


Supplementary Figure 5

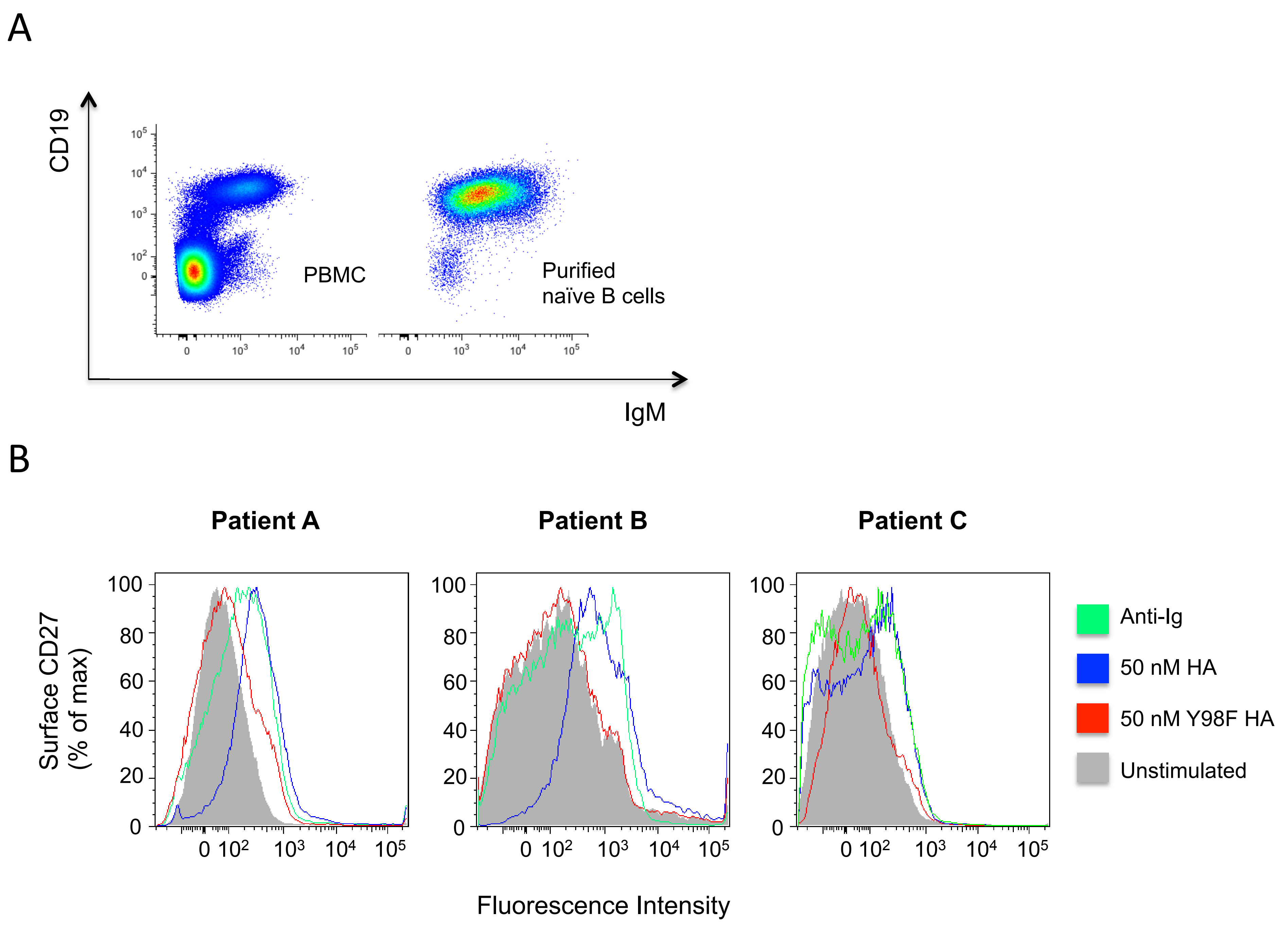


Supplementary Figure 6
